# Heart Failure in a Cohort of Patients with Chronic Kidney Disease: The GCKD Study

**DOI:** 10.1371/journal.pone.0122552

**Published:** 2015-04-13

**Authors:** Hanna Beck, Stephanie I. Titze, Silvia Hübner, Martin Busch, Georg Schlieper, Ulla T. Schultheiss, Christoph Wanner, Florian Kronenberg, Vera Krane, Kai-Uwe Eckardt, Anna Köttgen, GCKD Investigators

**Affiliations:** 1 Department of Medicine, Division of Nephrology, Medical Center—University of Freiburg, Freiburg, Germany; 2 Department of Nephrology and Hypertension, University of Erlangen-Nürnberg, Erlangen, Germany; 3 Department of Internal Medicine III, University of Jena, Jena, Germany; 4 Division of Nephrology and Clinical Immunology, Medical Faculty RWTH Aachen University, Aachen, Germany; 5 Department of Internal Medicine I, Division of Nephrology, University of Würzburg, Würzburg, Germany; 6 Comprehensive Heart Failure Centre, University of Würzburg, Würzburg, Germany; 7 Department of Medical Genetics, Molecular and Clinical Pharmacology, Division of Genetic Epidemiology, Medical University of Innsbruck, Innsbruck, Austria; Rouen University Hospital, FRANCE

## Abstract

**Background and Aims:**

Chronic kidney disease (CKD) is a risk factor for development and progression of heart failure (HF). CKD and HF share common risk factors, but few data exist on the prevalence, signs and symptoms as well as correlates of HF in populations with CKD of moderate severity. We therefore aimed to examine the prevalence and correlates of HF in the German Chronic Kidney Disease (GCKD) study, a large observational prospective study.

**Methods and Results:**

We analyzed data from 5,015 GCKD patients aged 18–74 years with an estimated glomerular filtration rate (eGFR) of <60 ml/min/1.73m^²^ or with an eGFR ≥60 and overt proteinuria (>500 mg/d). We evaluated a definition of HF based on the Gothenburg score, a clinical HF score used in epidemiological studies (Gothenburg HF), and self-reported HF. Factors associated with HF were identified using multivariable adjusted logistic regression. The prevalence of Gothenburg HF was 43% (ranging from 24% in those with eGFR >90 to 59% in those with eGFR<30 ml/min/1.73m2). The corresponding estimate for self-reported HF was 18% (range 5%-24%). Lower eGFR was significantly and independently associated with the Gothenburg definition of HF (*p*-trend <0.001). Additional significantly associated correlates included older age, female gender, higher BMI, hypertension, diabetes mellitus, valvular heart disease, anemia, sleep apnea, and lower educational status.

**Conclusions:**

The burden of self-reported and Gothenburg HF among patients with CKD is high. The proportion of patients who meet the criteria for Gothenburg HF in a European cohort of patients with moderate CKD is more than twice as high as the prevalence of self-reported HF. However, because of the shared signs, symptoms and medications of HF and CKD, the Gothenburg score cannot be used to reliably define HF in CKD patients. Our results emphasize the need for early screening for HF in patients with CKD.

## Introduction

Chronic kidney disease (CKD) is a major public health problem affecting more than 10% of the general population in many countries worldwide[1,2]. It is associated with high cardiovascular disease (CVD) morbidity and mortality[3,4,5]. CVD is often underdiagnosed and undertreated in patients with CKD[3]. Heart failure (HF) is one of the leading cardiovascular conditions in patients with impaired renal function[6]. CKD and HF occur frequently together[6,7,8], and epidemiological studies have found not only severely but already moderately reduced kidney function to be an independent risk factor for both incident HF[9,10,11] and aggravation of prevalent HF[12]. HF prevalence is estimated as 1–2% in the industrial states overall, 6–10% in people aged ≥65[13,14], and 40–54% in persons aged ≥65 who also have CKD[8].

In a recent report from the multi-ethnic Chronic Renal Insufficiency Cohort (CRIC), a US population of mostly middle-aged patients with CKD, 11% of patients reported previously diagnosed HF and an additional 25% reported symptoms of HF[15]. Corresponding data from patients with CKD of moderate severity are missing in Central Europe. The German Chronic Kidney Disease (GCKD) study is one of the largest prospective observational studies of referred CKD worldwide with 5217 Caucasian patients with CKD stage G3 or overt proteinuria enrolled from 2010–12[16].

There are no gold standard criteria to define HF in epidemiological studies, especially when the collection of echocardiogaphic data is not feasible. Many investigations use HF hospitalizations as a diagnosis criterion, which detects advanced disease but may miss patients with HF in early stages, where detection followed by efficient therapy can still prevent its progression. In this study, we aimed to investigate the prevalence, signs and symptoms as well as correlates of HF at the baseline visit of the GCKD study. In order to assess whether patients with moderate CKD already show signs and symptoms of HF found in its early stages, we evaluated the Gothenburg score, a validated clinical HF score that was developed to detect early HF [17,18,19]. We further evaluated a self-reported diagnosis of HF. The identification of HF, a frequent comorbidity in patients with CKD, or its clinical correlates represents an important first step towards improved management.

## Methods

### Study Population and Design

The GCKD study was designed as a prospective observational study of patients with moderate CKD in Germany to gain insights about the pathogenesis of CKD and its association with CVD. Main study outcomes include CKD progression, incidence of end-stage renal disease, cardiovascular events and mortality, and all-cause death. Details of the enrolment process and study procedures are fully described elsewhere[16]. Briefly, 5217 CKD patients under routine nephrological care were enrolled across nine German study centers. At the time of screening, male and female patients aged 18 to 74 years had an estimated glomerular filtration rate (eGFR) of 30–60 ml/min/1.73m^2^ (corresponding to the most prevalent CKD stage G3[20]), or overt proteinuria (albuminuria of >300 mg/g creatinine or proteinuria of >500 g/g creatinine) in the presence of higher eGFR. Exclusion criteria were non-Caucasian ethnicity, active malignancy, previous transplantations, HF stage NYHA IV, and legal attendance. All participants gave written informed consent, and the German Chronic Kidney Disease study was approved by the Ethics Committees of all participating institutions (Friedrich-Alexander-University Erlangen-Nuremberg, Medical Faculty of the Rheinisch-Westfälische Technische Hochschule Aachen, Charité—University Medicine Berlin, Medical Center—University of Freiburg, Medizinische Hochschule Hannover, Medical Faculty of the University of Heidelberg, Friedrich-Schiller-University Jena, Medical Faculty of the Ludwig-Maximilians-University Munich, Medical Faculty of the University of Würzburg).

### Data Collection

At the baseline visit, trained and certified personnel used standardized questionnaires to obtain information about the patient´s medical history, socio-demographic and life-style factors, medication intake and signs and symptoms of HF. The physical examination included measurements of body weight and height, heart rate and three measurements of resting blood pressure. Further information on the patients’ medical history as well as additional medical records were obtained from the patients’ treating nephrologists.

### Assessment of Heart Failure

The Gothenburg score[17,18,19] is a validated HF screening tool for epidemiological studies composed of three components: a cardiac score (positive if one or more of the following is present: coronary heart disease (CHD), angina pectoris, edema, dyspnea at night, pulmonary rales or atrial fibrillation), dyspnea on exertion, and the intake of HF medication. The presence of manifest HF defined as Gothenburg stage 2 (presence of a positive cardiac score with either dyspnea on exertion or HF medication) or stage 3 (presence of all three components) was evaluated. Pulmonary rales were not included since auscultation was not part of the physical exam. Developed in 1987, the original Gothenburg score defined digitalis or loop diuretics as typical HF medication. To allow for changes in treatment regimens over time, we included additional HF medication and established a modified definition that was used for the analyses in this report and was evaluated in several validation analyses (see below). HF according to the Gothenburg definition was compared to self-reported HF, which was assessed by asking the patients “Are you suffering from heart failure/weakness of the heart?”.

### Baseline Variables

All laboratory values were measured in a central certified laboratory using standardized protocols as described in detail previously[21]. Kidney function and damage was assessed by eGFR and the Urine Albumin-to-Creatinine Ratio (UACR) using the central laboratory measures from the baseline visit. GFR was estimated using the CKD-EPI formula[22] and categorized according to the KDIGO clinical practice guideline[23] into ≥90 (G1), 60-<90 (stage G2), 45-<60 (stage G3a), 30-<45 (stage G3b) and <30 (stage G4/5) ml/min/1.73m^2^. These values may differ from the screening values obtained from the treating nephrologists and resulted in the inclusion of some patients with an eGFR <30 ml/min/1.73m^2^. Differences in creatinine values between screening and baseline can be explained by the time difference between the screening and the baseline visit, and/or by differences in the creatinine assay and/or procedures between the central laboratory and the laboratories used by the treating nephrologists. UACR was calculated as measured urinary albumin/urinary creatinine (mg/g) and categorized according to the KDIGO classification[23] into <30, 30-<300 and ≥300 mg/g. Systolic and diastolic blood pressure (SBP and DBP (mmHg)) were determined as the average of up to three measured values. Hypertension was defined according to the Guidelines of the European Society of Hypertension and the European Society of Cardiology[24] as SBP ≥140 mmHg or DBP ≥90 mmHg or intake of antihypertensive medication. Diabetes mellitus was defined as HbA1c ≥6.5% or intake of antidiabetic medication according to the American Diabetes Society[25]. Anemia was defined as hemoglobin <12 g/dl (females) and <13 g/dl (males)[26]. Medication intake, edema, CHD, sleep apnea, angina pectoris, dyspnea on exertion, dyspnea at night, current smoking, alcohol consumption, chronic obstructive pulmonary disease (COPD), asthma and educational level were obtained by standardized interviews. Atrial fibrillation (chronic or intermittent) and valvular heart disease were obtained by interviewing the treating nephrologist. CHD was considered present when the participant had ever had a heart attack or undergone a coronary reperfusion procedure (bypass or angioplasty). Angina pectoris was classified by the Rose questionnaire[27]. Medication intake was programmed using ATC-Codes[28]. Educational level was categorized into ≤9, 10 and >10 years according to the different German school-types.

### Validation Analyses

To examine the use of the Gothenburg score in a CKD population in more detail, several additional analyses were conducted. A subgroup analysis of the GCKD participants enrolled at the Freiburg center (541 patients) was performed; plausibility checks were conducted in order to ensure a representative sample. Additional medical records supplied by the treating nephrologists or from former hospitalizations were abstracted and used to identify 118 patients with data on HF and/or echocardiographic examinations, 29 of whom had an echo-based and/or clinically established HF diagnosis based on the medical abstraction (diagnosed acute or chronic HF, cardiac decompensation, dilatative cardiomyopathy, or ICD-10 code for HF). Among these 118 patients, sensitivity, specificity, positive predictive value (PPV) and negative predictive value (NPV) were calculated comparing the prevalence of HF based on different definitions (various Gothenburg score modifications and self-reported HF) to the prevalence of abstracted HF. Gothenburg score modifications consisted of different combinations of current guideline-based HF medication[29] including angiotensin-converting enzyme (ACE) inhibitors, ARBs, β-blockers, mineralocorticoid receptor antagonists (MRA), loop diuretics, digitalis and ivabradine. The combination of ACE inhibitors and either digitalis or loop diuretics or ivabradine showed the best results in these analyses and was subsequently used to establish the modified Gothenburg score used in this report.

Additional analyses were conducted evaluating patients that were recruited for low eGFR separately from those recruited for high proteinuria, as well as analyses restricting the definition of manifest HF to Gothenburg stage 3.

### Statistical Analyses

All analyses were conducted using STATA 12 (College Park, Texas). Analyses were limited to 5015 participants excluding those who were missing information needed to calculate eGFR or UACR and to generate the Gothenburg score (see S1 Fig). Factors associated with prevalent HF were selected for evaluation based on previously reported HF risk factors, listed as covariates above. Descriptive statistics comparing risk factors between individuals with and without prevalent HF were generated using t-tests for continuous, chi-squared tests for categorical variables and Wilcoxon rank-sum test for UACR. Adjusted risk factor associations for the presence of Gothenburg HF and self-reported HF were obtained from multivariable adjusted logistic regression models including pre-specified covariates of interest and study center. Variables that were part of the Gothenburg score, e.g. CHD and atrial fibrillation, were not included in the multivariable model. Two-sided p-values of <0.05 were considered statistically significant. Model fit was examined using goodness of fit tests. Associated covariates were compared for the direction and magnitude of their association across the different definitions of HF. Effect modifications of the association between eGFR and HF by UACR, sex, and diabetes were evaluated by including the corresponding interaction terms into the regression models. To account for potential confounders, stratified analyses were conducted by absence or presence of CHD, asthma or COPD.

## Results

### Study population and baseline characteristics

Characteristics of the study population by eGFR categories are presented in Table 1; the majority of patients had CKD stage G3a (33%) or stage G3b (36%). Consistent with the GCKD recruitment strategy, the median UACR was high in individuals with early-stage CKD (stage G1). The proportion of patients with CHD and diabetes mellitus was higher with more advanced CKD: from 4% (stage G1) to 29% (stage G4/5) for CHD and from 21% to 45% for diabetes mellitus.

**Table 1:** Characteristics of 5015 GCKD study participants by eGFR categories.

	**eGFR categories (ml/min/1.73 m** ^**2**^ **)**				
Characteristics	≥90 (n = 232; 4.6%) CKD stage G1	60–89 (n = 860; 17.2%) CKD stage G2	45–59 (1663; 33.2%) CKD stage G3a	30–44 (n = 1815; 36.2%) CKD stage G3b	<30 (n = 445; 8.9%) CKD stage G4/5
**UACR (mg/g): median (IQR)**	531.3 (165.1, 1171.2)	43.5 (7.8, 441.0)	26.1 (6.8, 191.3)	57.6 (11.5, 352.5)	122.9 (23.2, 905.3)
**UACR categories (mg/g)**					
** < 30**	12.1 (28)	45.0 (387)	52.0 (865)	40.8 (740)	29.0 (129)
** 30–299**	22.0 (51)	25.7 (221)	27.7 (461)	32.1 (582)	32.4 (144)
** ≥300**	66.0 (153)	29.3 (252)	20.3 (337)	27.2 (493)	38.7 (172)
**Age (years)**	41.9 (13.1)	55.7 (12.5)	61.4 (10.3)	62.6 (10.6)	63.5 (10.0)
**BMI (kg/m^2^)**	28.6 (6.7)	29.3 (5.9)	29.8 (5.8)	30.1 (6.0)	30.5 (6.2)
**Male gender**	52.2 (121)	54.4 (468)	61.9 (1030)	61.4 (1115)	62.9 (280)
**Diabetes mellitus**	21.1 (49)	27.8 (239)	35.1 (583)	38.1 (692)	44.9 (200)
**Hypertension**	91.8 (213)	90.5 (778)	94.7 (1574)	97.1 (1762)	98.4 (438)
**Coronary heart disease**	3.5 (8)	13.0 (112)	19.5 (325)	23.3 (422)	28.5 (127)
**Atrial fibrillation**	2.6 (6)	6.1 (52)	8.2 (136)	11.5 (209)	13.1 (58)
**Valvular heart disease**	1.3 (3)	8.3 (71)	8.6 (142)	11.7 (211)	12.4 (55)
**Sleep apnea**	4.7 (11)	8.0 (69)	10.1 (168)	9.5 (173)	14.2 (63)
**Anemia**	12.7 (28)	14.6 (122)	18.1 (292)	28.9 (511)	45.9 (196)
**Hemoglobin (g/dl)**	14.04 (1.99)	13.91 (1.83)	13.81 (1.65)	13.38 (1.78)	12.76 (1.61)
**Serum Albumin (g/l)**	36.2 (5.9)	38.2 (4.8)	38.8 (4.0)	38.4 (4.0)	37.5 (4.1)
**Heart rate (bpm)**	74.3 (10.3)	71.1 (12.5)	70.2 (11.8)	69.9 (12.3)	70.5 (12.5)
**Current smoker**	34.9 (81)	18.6 (159)	14.0 (232)	14.7 (266)	14.4 (64)
**Alcohol intake (≥ 3 times per week)**	14.7 (34)	20.4 (174)	21.2 (352)	17.1 (308)	17.9 (79)
**Education**					
** ≤9 years**	32.6 (73)	46.3 (388)	52.6 (857)	60.5 (1080)	64.0 (281)
** 10 years**	32.6 (73)	32.6 (273)	31.1 (506)	24.9 (445)	23.7 (104)
** >10 years**	34.8 (78)	21.1 (177)	16.3 (265)	14.6 (260)	12.3 (54)

Data are mean (SD) for continuous variables and percentages (count) for categorical variables. Missing values in following variables (number of missings): BMI (57), atrial fibrillation (12), valvular heart disease (42), anemia & hemoglobin (145), serum albumin (1), heart rate (49), current smoker (12), alcohol intake (28), education (101).

Valvular heart disease: aortic stenosis (n = 73), aortic insufficiency (n = 142), mitral stenosis (n = 15), mitral insufficiency (n = 251), other (n = 169). Some individuals had more than one type of valvular heart disease.

The characteristics of the GCKD study population by presence and absence of Gothenburg or self-reported HF are compared in S1 Table. In these univariate analyses, eGFR and UACR were lower and BMI was higher in patients with Gothenburg HF compared to those without HF. Moreover, patients with Gothenburg HF were older, had more diabetes mellitus, CHD, atrial fibrillation, valvular heart disease, anemia and sleep apnea, and had lower educational attainment than patients without Gothenburg HF. Self-reported HF demonstrated similar relationships.

### Prevalence of heart failure

The prevalence of HF when applying the Gothenburg criteria was 43% (27% Gothenburg stage 2 and 16% Gothenburg stage 3), compared to 18% for self-reported HF. Of patients with self-reported HF, 79% were also classified as having Gothenburg HF. The exact composition of the Gothenburg score and the proportion of GCKD patients in each of its components are displayed in Table 2. Edema was the most common of the score components with 40%, followed by CHD. S2 Table shows the components of the Gothenburg score separately for men and women. A higher proportion of women reported edema and/or dyspnea on exertion, whereas a higher proportion of men reported a history of coronary heart disease and/or the intake of HF medication.

**Table 2:** Composition of the Gothenburg score and proportions of GCKD participants within the cardiac score components.

**Gothenburg stage**	**Symptoms and findings**	**GCKD cohort**
0: No HF	No history, no signs, no HF medication	42%
1: Latent HF	Cardiac score ≥ 1	15%
2: Manifest HF	Cardiac score ≥ 1 and dyspnea on exertion or	17%
	Cardiac score ≥ 1 and HF medication	10%
3: Manifest HF	Cardiac score ≥ 1, dyspnea on exertion and HF medication	16%
**Cardiac score**	**Points ≥ 1**	**58%**
Coronary heart disease	1	20%
Angina pectoris	1	9%
Edema	1	40%
Dyspnea at night	1	13%
Atrial fibrillation	1	9%
Pulmonary rales	1	Not obtained in GCKD	

**Fig 1:**
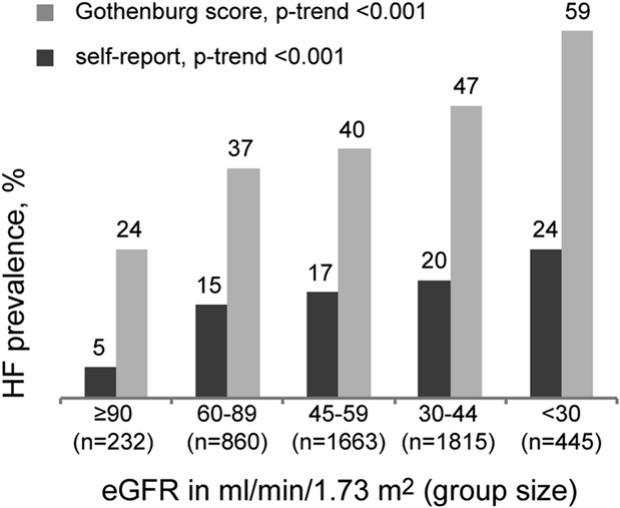
Prevalence of heart failure across eGFR categories. The prevalence of both self-reported and Gothenburg score heart failure is higher with lower eGFR category, with Gothenburg heart failure observed at least twice as much in each category compared to self-report. P-trend was determined from logistic regressions of each heart failure definition on categorized eGFR.

Fig 1 shows that the prevalence of both Gothenburg and self-reported HF was significantly higher among individuals in lower eGFR categories (*p*-trend for both <0.001). The prevalence of Gothenburg HF rose from 24% in CKD stage G1 to 59% in stage G4/5, whereas the prevalence of self-reported HF rose from 5% in CKD stage G1 to 24% in CKD stage G4/5.

Table 3 shows the prevalence of both HF definitions across different UACR categories and separately among men and women as well as among individuals with and without diabetes mellitus and CHD. HF according to the Gothenburg score showed the highest prevalence (47%) among patients with normoalbuminuria (<30 mg/g) and similar prevalences (40 and 39%) among patients with micro- and macroalbuminuria (30-<300 and ≥300 mg/g). The prevalence of self-reported HF decreased with increasing UACR in these univariate analyses. Gothenburg HF was more prevalent in female than in male patients (46% versus 41%), whereas there was no appreciable sex difference in the prevalence of self-reported HF. Both HF definitions showed a substantially higher prevalence in diabetic patients compared to non-diabetic patients (59% vs. 34% for Gothenburg HF and 26% vs. 13% for self-reported HF) and in patients with CHD compared to patients without CHD (79% vs. 34% for Gothenburg HF and 43% vs. 11% for self-reported HF).

**Table 3:** Prevalence of heart failure in UACR categories, by gender, presence of diabetes mellitus and CHD.

	**Gothenburg score**	**self-report**
**Overall (n = 5015)**	42.8 (2146)	17.7 (889)
**UACR categories (mg/g)**		
** <30 (n = 2149)**	46.9 (1008)	21.3 (457)
** 30–299 (n = 1459)**	40.0 (584)	17.6 (257)
** ≥300 (n = 1407)**	39.4 (554)	12.4 (175)
**p-value**	<0.001	<0.001
**Female (n = 2001)**	45.6 (913)	16.9 (338)
**Male (n = 3014)**	40.9 (1233)	18.3 (551)
**p-value**	0.001	0.207
**Non-diabetic (n = 3252)**	34.3 (1115)	13.2 (429)
**Diabetic (n = 1763)**	58.5 (1031)	26.1 (460)
**p-value**	<0.001	<0.001
**No CHD (n = 4021)**	34.0 (1365)	11.4 (459)
**CHD (n = 994)**	78.6 (781)	43.3 (430)
**p-value**	<0.001	<0.001

Data are percentages (count). P-values are provided for a comparison of characteristics within a given definition of HF, e.g. proportion of men and women with self-reported HF.

### Validation analyses

Twenty-five percent of the 118 patients with information on HF and/or echocardiographic examinations had a HF diagnosis. Compared to this information, the modified Gothenburg score showed high sensitivity (83%) and a high negative predictive value (91%), moderate specificity of 55% and a low positive predictive value of 38% (Table 4); the original Gothenburg score showed similar results. Self-reported HF showed lower sensitivity (52%) and higher specificity (89%). Additional sensitivity analyses evaluating patients that were recruited for low eGFR and those recruited for high proteinuria led to similar results; the evaluation of other medication combinations including ß-blockers and MRAs and only counting Gothenburg stage 3 as manifest HF did not show higher measures of validity.

**Table 4:** Validation analyses within a subsample of the regional center Freiburg (n = 118).

	**Sensitivity**	Specificity	PPV	NPV
**Gothenburg (modified definition)**	83	55	38	91
**Gothenburg (original definition)**	83	54	38	91
**Self-reported heart failure**	52	89	60	85

Data are percentages.

Reference is heart failure diagnosis based on abstraction of medical records of the treating nephrologists or former hospitalizations. Sensitivity is the proportion of patients with heart failure according to the respective evaluated definition out of the patients with heart failure according to the reference definition. Specificity is the proportion of patients without heart failure according to the respective definition out of the patients without heart failure according to the reference definition. Positive predictive value (PPV) is the proportion of patients with heart failure according to the reference definition out of the patients with heart failure according to the evaluated definition. Negative predictive value (NPV) is the proportion of patients without heart failure according to the reference definition out of the patients without heart failure according to the evaluated definition.

### Factors associated with heart failure

Table 5 presents factors associated with one or both HF definitions obtained from a multivariate adjusted logistic regression model. Many risk factor associations were statistically significant and of similar magnitude across the two HF definitions, but some factors showed differences. Gothenburg HF was significantly associated with lower eGFR: compared with individuals in CKD stage G3a, the odds ratio of Gothenburg HF among individuals with stage G3b CKD was 1.18 (95% CI 1.01–1.39), and 1.71 among those with stage G4/5 CKD (95% CI 1.32–2.20). In comparison with individuals with normoalbuminuria, microalbuminuria was inversely associated with prevalent Gothenburg HF (OR 0.81, 95% CI 0.69–0.95), whereas the association of macroalbuminuria was not significant. In contrast, prevalent self-reported HF was not significantly associated with eGFR-categories although the odds ratios also increased with lower eGFR, and was inversely associated with macroalbuminuria but not microalbuminuria. Adjustment for ACE inhibitor or ARB intake did not change the association between albuminuria and Gothenburg or self-reported HF. Male gender was inversely associated with Gothenburg HF (OR 0.65, 95% CI 0.57–0.76), whereas there was no significant association with self-reported HF. There were no significant interactions between category of eGFR and UACR, diabetes or sex for either definition of HF (all p>0.3).

**Table 5:** Multivariable adjusted analyses of factors associated with Gothenburg HF and self-reported HF (n = 4,604).

	**Gothenburg HF**			Self-reported HF		
	OR	95% CI	*P*	OR	95% CI	*P*
**eGFR (ml/min/1.73 m^2^)**						
** ≥90**	0.87	0.59–1.29	0.049	0.56	0.27–1.14	0.111
** 60–89**	1.07	0.88–1.31	0.485	1.16	0.90–1.49	0.264
** 45–59 (reference)**	ref	ref	ref	ref	ref	ref
** 30–44**	1.18	1.01–1.39	0.036	1.12	0.92–1.36	0.253
** <30**	1.71	1.32–2.20	<0.001	1.29	0.96–1.74	0.088
**UACR (mg/g) (reference: <30)**	ref	ref	ref	ref	ref	ref
** 30–299**	0.81	0.69–0.95	0.010	0.87	0.71–1.05	0.147
** ≥300**	0.87	0.72–1.04	0.126	0.70	0.55–0.88	0.002
**Age (5 year intervals)**	1.13	1.09–1.17	<0.001	1.15	1.10–1.22	<0.001
**Male gender**	0.65	0.57–0.76	<0.001	1.01	0.84–1.21	0.930
**Diabetes mellitus**	1.64	1.42–1.90	<0.001	1.61	1.35–1.92	<0.001
**Hypertension**	1.81	1.27–2.59	0.001	1.90	1.11–3.26	0.020
**Valvular heart disease**	2.52	2.01–3.15	<0.001	3.98	3.19–4.97	<0.001
**BMI (kg/m^2^)**	1.10	1.09–1.11	<0.001	1.04	1.03–1.06	<0.001
**Sleep apnea**	2.22	1.76–2.81	<0.001	2.15	1.70–2.73	<0.001
**Anemia**	1.39	1.18–1.63	<0.001	1.06	0.88–1.29	0.534
**Education (reference: ≤9 years)**	ref	ref	ref	ref	ref	ref
** 10 years**	0.84	0.72–0.99	0.037	0.94	0.77–1.15	0.534
** >10 years**	0.69	0.56–0.84	<0.001	1.00	0.78–1.29	0.977
**Serum albumin (g/l)**	0.95	0.94–0.97	<0.001	0.99	0.98–1.02	0.993
**Heart rate (bmp)**	0.99	0.99–1.00	0.584	0.99	0.98–0.99	0.011
**Current smoker**	0.97	0.80–1.17	0.752	0.98	0.76–1.26	0.864
**Alcohol intake (≥ 3 times per week)**	0.98	0.82–1.17	0.827	0.97	0.79–1.21	0.802

Of 5,015 observations, values were missing values in BMI (57), valvular heart disease (42), anemia (145), serum albumin (1), education (101), heart rate (49), current smoker (12), alcohol intake (28).

To evaluate if the strong associations with Gothenburg HF would persist when excluding patients with self-reported HF, we conducted a multivariate logistic regression for the 3,789 patients without self-reported HF and with full covariate information (S3 Table). The association of both stage G3b and stage G4/5 CKD with Gothenburg HF remained essentially unchanged (OR 1.18 and 1.85, respectively) although significance was attenuated due to smaller sample size. All other associations remained significantly associated with similar odds ratios.

We conducted a number of further analyses to assess whether additional confounders were likely to be important. To explore if the GCKD recruitment strategy (inclusion for low eGFR vs. high proteinuria) had an influence on the associations of UACR with HF, we performed the multivariate analysis only for patients that had been recruited based on eGFR<60 ml/min/1.73m^2^ and had full covariate information (*n* = 4,278) and observed very similar results (S4 Table). Secondly, asthma and COPD as potential alternative causes for dyspnea on exertion were included into the multivariate model with similar results. In addition, stratifying the patients for the presence or absence of asthma and of COPD also did not alter the associations observed above. Lastly, the inclusion of CHD into the multivariate model showed a highly significant association with both Gothenburg (OR 6.30, 95% CI 5.19–7.64) and self-reported HF (OR 4.42, 95% CI 3.67–5.33) and led to a slight attenuation of some of the other associations, but all remained highly significant. However, since CHD is one of the variables on which the Gothenburg score is based, it was not included in the primary model testing risk factor associations with prevalent HF.

## Discussion

In this cross-sectional analysis of a German CKD population, 43% met the criteria of the Gothenburg HF score, and 18% self-reported the presence of HF. The prevalence of HF from both definitions was significantly higher among individuals with lower eGFR. These observations show that a large fraction of patients with moderately severe CKD shows signs and symptoms of HF although many of the patients are not aware of a HF diagnosis.

Only few studies have conducted cross-sectional epidemiological investigations that address the prevalence, signs and symptoms and correlates of HF in patients with CKD, and our study is the first to explore these questions in a large European CKD cohort. A recent investigation in the CRIC Study showed that in a US population of patients with CKD, 11% reported previously diagnosed HF and an additional 25% reported symptoms of HF. The authors concluded that those symptoms likely indicate the presence of early HF in many of the participants[15]. The HF symptoms evaluated in the CRIC Study included dyspnea and edema, which were also part of the Gothenburg score used in our analyses. The proportion of patients with HF as defined by the Gothenburg score was even higher in GCKD, which might be due to the fact that the Gothenburg score incorporates also medication intake as well as due to the exclusion of advanced HF stages (NYHA III and IV) in CRIC, whereas only individuals with HF NYHA IV were excluded from GCKD. Another investigation of an older population (Medicare patients ≥65 years) with non-dialysis-dependent CKD showed a prevalence of advanced HF identified through hospitalization codes of 40% among non-diabetic and 54% among diabetic patients[8]. In an analysis of the population-based NHANES survey, prevalent self-reported HF rose from 7% in patients with CKD G3b to 28% in stage G4[30]. The high prevalence of HF or of its signs and symptoms observed in our study is thus supported by similar estimates from other studies.

We observed a strong association between lower eGFR and HF, which remained significant for Gothenburg HF after multivariate adjustment. This is consistent with other studies that have shown an independent association between lower eGFR and HF[9,11] as well as general CVD morbidity and mortality risk[5]. Contrary to some reports[5] that have shown increased albuminuria to be associated with CVD, our multivariable adjusted analyses did not identify a significant association between increased albuminuria and HF, but in fact an inverse association between albuminuria and HF. To explore whether this observation might be related to the recruitment strategy in the GCKD study, we conducted multivariable adjusted analyses restricted to 4,278 individuals recruited based on eGFR <60 ml/min/1.73m^2^ and full covariate information (S4 Table). The inverse association between increased albuminuria and HF persisted, which suggests that the GCKD recruitment scheme is not sufficient to explain the absence of the previously reported association of higher UACR with HF. A potential explanation that may contribute to differences observed to previous population-based samples is the high intake of medications that impact the UACR in a CKD population.

Risk factor associations with HF were evaluated with a focus on previously reported factors and comorbidities[14]. Older age, higher BMI, the presence of hypertension, diabetes mellitus, valvular heart disease, sleep apnea, and anemia as well as lower educational attainment were significantly associated with prevalent HF. Generally, males have been reported to be at higher risk to develop HF, especially HF with reduced ejection fraction and of ischemic etiology[31]. Our analyses revealed no association between male sex and self-reported HF, but a significant inverse association between male sex and the Gothenburg definition of HF. A potential explanation for this observation could be a high proportion of patients with HF with preserved ejection fraction (HF-PEF) in the GCKD Study, which is more common in females and patients with hypertension[14,31,32]. This hypothesis is supported by the observation that left ventricular diastolic dysfunction is frequent among patients with CKD[33]. In addition, awareness may be lower in patients with HF-PEF than in patients with HF with reduced ejection fraction, which is associated with male sex and ischemic etiology[14,31]. Together, these observations could explain that—in contrast to self-reported HF—the Gothenburg score is associated with female sex in our study. Another explanation of the higher prevalence of Gothenburg HF in women may be the higher proportion of women reporting edema, which other than from HF or CKD could also result from venous circulation disorders that are more commonly observed in women.[34] The stronger association between lower eGFR and Gothenburg HF compared to self-reported HF could be influenced by a more sensitive definition of Gothenburg HF, or by the lower specificity leading to the inclusion of more patients into the Gothenburg definition where signs and symptoms of HF were in fact due to CKD rather than HF.

The association between HF and CKD is bi-directional. A recent investigation of the ESC Heart Failure Pilot survey has demonstrated that in a cohort of patients with chronic HF, CKD was prevalent in 41% and was independently associated with a higher risk of mortality and HF hospitalization[35]. The contribution of heart diseases to worsening renal function and vice versa have been described as cardio-renal-syndromes[36]. The association between CKD and HF/CVD can only partly be explained by common risk factors and comorbidities like older age, hypertension, diabetes mellitus and atherosclerosis; independent associations between a decline in renal function and CVD/HF have been found[3,4,5].

The Gothenburg score is a HF screening tool that was developed to detect HF already in its early stages in epidemiological studies and to differentiate between cardiac and pulmonary causes of dyspnea[17,18,19]. A study in a community-based population reported a sensitivity of 84% and specificity of 81%, compared to the gold standard definition of HF based on the Guidelines of the European Society of Cardiology[37]. Our study is the first to investigate the use of the Gothenburg score in a cohort of CKD patients. In our subgroup analysis, most of the individuals with a HF diagnosis based on echocardiography were captured by the Gothenburg HF definition (sensitivity of 83%). The negative predictive value of 91% supports that individuals who do not meet the Gothenburg score definition of HF do in fact not suffer from diagnosed HF, which will be useful to determine the incidence of HF in future longitudinal analyses of the GCKD cohort where patients with prevalent HF have to be excluded. The moderate specificity of 55% and the low positive predictive value of 38% indicate that Gothenburg HF definition contains false positives but its high NPV indicates its potential usefulness as a screening test to identify patients to undergo echocardiographic examinations. It needs however be taken into account that the echocardiographical examinations had been performed upon some indication rather than among everyone, such that early forms of HF and HF with preserved ejection fraction might have been underdiagnosed.

A likely alternative explanation for the false positive assignment of HF based on the Gothenburg score might be that the symptoms and signs used for the composition of the Gothenburg score cannot uniquely be attributed to HF but could also result from other diseases, e.g. dyspnea from pulmonary diseases or because of obesity. Adjustment for these potential confounders—obesity, asthma and COPD—did however not alter the observed results. Moreover, edema, which is also part of the Gothenburg score, is a typical sign of both HF and CKD and was present in 40% of the GCKD cohort. Furthermore the medication that is part of the Gothenburg score definition is not specific for HF but could also have been prescribed for other reasons, e.g. ACE inhibitors for hypertension or proteinuria, but it is not possible to obtain information about the indication of prescriptions in epidemiological studies. Taken together, the Gothenburg score cannot be used to reliably define HF in CKD patients, because both diseases share signs, symptoms and medications. Nevertheless, the high prevalence of HF signs and symptoms in CKD patients warrants a cardiologic evaluation of these patients for the presence of HF.

Our results have several clinical implications. Because both CKD and HF can lead to hypervolemia, it is difficult for physicians to detect HF in patients with CKD. However, assessing the presence of HF and improving awareness of the condition as a frequent comorbidity in patients with CKD represents an important first step towards early initiation of therapeutic management. This should include consequent HF monitoring and treatment as well as attention to and treatment of common risk factors like hypertension, diabetes mellitus, obesity and anemia that can contribute to the worsening of both HF and CKD. Because some medications such as ACE inhibitors are prescribed both for HF and for CKD, patients using ACE inhibitors may have a therapeutic benefit even if HF is not diagnosed. Both CKD and HF are global public health problems increasing with older age. Consequently the prevalence of both diseases separately or concomitantly and their associated costs will rise even more in the future in aging industrial populations.

Several limitations warrant mention: first, it was not feasible to conduct echocardiographic examinations as part of the GCKD visits, since the exams took place at many different sites precluding the use of comparable devices and protocols. However, data from echocardiographic examinations conducted outside the study visits that were available in the medical records of a sub-cohort were used for validation analyses. The Gothenburg score is based on medications and signs and symptoms of disease that may result both from HF and CKD as well as their combination, such that in a CKD population, the score can be used to quantify the presence of these signs and symptoms but not to diagnose HF. Moreover, sufficient information to be able to differentiate between acute decompensated HF and chronic stable HF was not available. This shortcoming of the Gothenburg score has been observed by others in epidemiological settings[38]. Second, the available data are cross-sectional and thus do not allow to infer temporal relationships. Third, residual confounding of associations through unknown risk factors may remain. Fourth, we examined a homogeneous cohort of German CKD patients under nephrological care, so they may not be generalizable to CKD in other settings or more advanced stages of the disease. Notable strengths of our study include its large sample size, which ensures high statistical power, the use of standardized questionnaires and in-person study visits conducted by trained study nurses, which reduces heterogeneity, the collection of detailed medication information, and reliable estimation of kidney function based on central measurements of all laboratory values.

In summary, use of the Gothenburg score to assess HF in a European cohort of patients with moderately severe CKD leads to prevalence estimates that are more than twice as high as those for self-reported HF. Lower eGFR was a significant independent correlate of Gothenburg HF. Because of the shared signs, symptoms and medications of HF and CKD, the Gothenburg score is not useful to reliably define HF in the setting of CKD, but our results emphasize the need to evaluate CKD patients with HF signs and symptoms for the presence of HF as a first step for its early identification and treatment.

## Supporting Information

S1 Fig Selection of the Study Sample. (DOCX)

S1 Table Characteristics of the GCKD study population (n = 5015) by heart failure. (DOCX)

S2 Table Composition of the Gothenburg score in the GCKD study stratified by gender. (DOCX)

S3 Table Multivariable adjusted analyses of factors associated with Gothenburg HF among patients without self-reported HF (n = 3,789). (DOCX)

S4 Table Multivariable adjusted analysis of factors associated with HF restricted to patients recruited because of an eGFR <60 ml/min/1.73m^2^ (n = 4,278). (DOCX)
