# Correction: Heart Failure in a Cohort of Patients with Chronic Kidney Disease: The GCKD Study

**DOI:** 10.1371/journal.pone.0131034

**Published:** 2015-06-15

**Authors:** Hanna Beck, Stephanie I. Titze, Silvia Hübner, Martin Busch, Georg Schlieper, Ulla T. Schultheiss, Christoph Wanner, Florian Kronenberg, Vera Krane, Kai-Uwe Eckardt, Anna Köttgen, GCKD Investigators

There is an error in Table 5 in the third column’s second row. The correct p-value should be 0.495. Please see the corrected Table 5 here.

**Table 5:** Multivariable adjusted analyses of factors associated with Gothenburg HF and self-reported HF (n = 4,604)

	**Gothenburg HF**			**Self-reported HF**		
	OR	95% CI	*P*	OR	95% CI	*P*
**eGFR (ml/min/1.73 m** ^**2**^ **)**						
** ≥90**	0.87	0.59–1.29	0.495	0.56	0.27–1.14	0.111
** 60–89**	1.07	0.88–1.31	0.485	1.16	0.90–1.49	0.264
** 45–59 (reference)**	ref	ref	ref	ref	ref	ref
** 30–44**	1.18	1.01–1.39	0.036	1.12	0.92–1.36	0.253
** <30**	1.71	1.32–2.20	<0.001	1.29	0.96–1.74	0.088
**UACR (mg/g) (reference: <30)**	ref	ref	ref	ref	ref	ref
** 30–299**	0.81	0.69–0.95	0.010	0.87	0.71–1.05	0.147
** ≥300**	0.87	0.72–1.04	0.126	0.70	0.55–0.88	0.002
**Age (5 year intervals)**	1.13	1.09–1.17	<0.001	1.15	1.10–1.22	<0.001
**Male gender**	0.65	0.57–0.76	<0.001	1.01	0.84–1.21	0.930
**Diabetes mellitus**	1.64	1.42–1.90	<0.001	1.61	1.35–1.92	<0.001
**Hypertension**	1.81	1.27–2.59	0.001	1.90	1.11–3.26	0.020
**Valvular heart disease**	2.52	2.01–3.15	<0.001	3.98	3.19–4.97	<0.001
**BMI (kg/m** ^**2**^ **)**	1.10	1.09–1.11	<0.001	1.04	1.03–1.06	<0.001
**Sleep apnea**	2.22	1.76–2.81	<0.001	2.15	1.70–2.73	<0.001
**Anemia**	1.39	1.18–1.63	<0.001	1.06	0.88–1.29	0.534
**Education (reference: ≤9 years)**	ref	ref	ref	ref	ref	ref
** 10 years**	0.84	0.72–0.99	0.037	0.94	0.77–1.15	0.534
** >10 years**	0.69	0.56–0.84	<0.001	1.00	0.78–1.29	0.977
**Serum albumin (g/l)**	0.95	0.94–0.97	<0.001	0.99	0.98–1.02	0.993
**Heart rate (bmp)**	0.99	0.99–1.00	0.584	0.99	0.98–0.99	0.011
**Current smoker**	0.97	0.80–1.17	0.752	0.98	0.76–1.26	0.864
**Alcohol intake (≥ 3 times per week)**	0.98	0.82–1.17	0.827	0.97	0.79–1.21	0.802

Of 5015 observations, values were missing in BMI (57), valvular heart disease (42), anemia (145), serum albumin (1), education (101), heart rate (49), current smoker (12), alcohol intake (28).
