# Supplementary figures and images for: Heart Failure in a Cohort of Patients with Chronic Kidney Disease: The GCKD Study
Source: PLoS One. 2015 Apr 13;10(4):e0122552. doi: 10.1371/journal.pone.0122552 (PMC4395150; doi:10.1371/journal.pone.0122552)

**S1 Figure: Selection of the Study Sample**

**
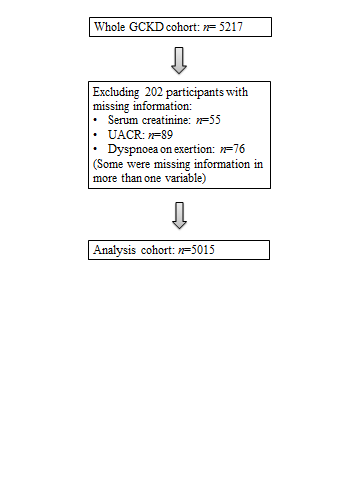
**

Supplement: S1 Fig — (DOCX) [file pone.0122552.s001.docx]
