# Supplementary material for: Heart Failure in a Cohort of Patients with Chronic Kidney Disease: The GCKD Study
Source: PLoS One. 2015 Apr 13;10(4):e0122552. doi: 10.1371/journal.pone.0122552 (PMC4395150; doi:10.1371/journal.pone.0122552)
Supplement: S1 Table — (DOCX) [file pone.0122552.s002.docx]

**S1 Table: Characteristics of the GCKD Study Population (n=5015) by Heart Failure**

|  | Gothenburg score | | |  | self-report |  |  |
| --- | --- | --- | --- | --- | --- | --- | --- |
| Characteristics | **no HF (n=2869; 57.2%)** | | **HF (n=2146; 42.8%)** | ***P*** | **no HF (n=4126, 82.3%)** | **HF (n=889, 17.7%)** | ***P*** |
| eGFR (ml/min/1.73 m²) | 51.8 (19.1) | 46.4 (16.6) | | <0.001 | 50.3 (18.8) | 45.6 (14.8) | <0.001 |
| eGFR categories (CKD-EPI) | |  | | <0.001 |  |  | <0.001 |
| ≥90 | 6.1 (176) | 2.6 (56) | |  | 5.4 (221) | 1.2 (11) |  |
| 60-89 | 18.9 (543) | 14.8 (317) | |  | 17.7 (730) | 14.6 (130) |  |
| 45-59 | 35.1 (1006) | 30.6 (657) | |  | 33.4 (1379) | 32.0 (284) |  |
| 30-44 | 33.5 (962) | 39.8 (853) | |  | 35.3 (1456) | 40.4 (359) |  |
| <30 | 6.3 (182) | 12.3 (263) | |  | 8.2 (340) | 11.8 (105) |  |
| UACR (mg/g): median (IQR) | 60.4 (10.8, 420.5) | 38.4 (8.6, 326.7) | | <0.001 | 58.1 (10.1, 445.5) | 28.2 (8.0, 157.5) | <0.001 |
| UACR categories (mg/g) | |  | | <0.001 |  |  | <0.001 |
| < 30 | 39.8 (1141) | 47.0 (1008) | |  | 41.0 (1692) | 51.4 (457) |  |
| 30-299 | 30.5 (875) | 27.2 (584) | |  | 29.1 (1202) | 28.9 (257) |  |
| ≥300 | 29.7 (853) | 25.8 (554) | |  | 29.9 (1232) | 19.7 (175) |  |
| Age (years) | 57.9 (12.9) | 63.1 (9.7) | | <0.001 | 59.1 (12.3) | 64.7 (8.4) | <0.001 |
| BMI (kg/m²) | 28.1 (5.2) | 32.0 (6.2) | | <0.001 | 29.4 (5.8) | 31.7 (6.3) | <0.001 |
| Male gender | 62.1 (1781) | 57.5 (1233) | | 0.001 | 59.7 (2463) | 62.0 (551) | 0.207 |
| Diabetes mellitus | 25.5 (732) | 48.0 (1031) | | <0.001 | 31.6 (1303) | 51.7 (460) | <0.001 |
| Hypertension | 93.2 (2674) | 97.4 (2091) | | <0.001 | 94.4 (3894) | 98.0 (871) | <0.001 |
| Coronary heart disease | 7.4 (213) | 36.4 (781) | | <0.001 | 13.7 (564) | 48.4 (430) | <0.001 |
| Atrial fibrillation | 2.9 (82) | 17.7 (379) | | <0.001 | 5.7 (233) | 25.7 (228) | <0.001 |
| Valvular heart disease | 6.2 (176) | 14.4 (306) | | <0.001 | 6.7 (274) | 23.6 (208) | <0.001 |
| Sleep apnea | 5.3 (152) | 15.5 (332) | | <0.001 | 7.7 (318) | 18.7 (166) | <0.001 |
| Anemia | 20.1 (562) | 28.2 (587) | | <0.001 | 23.0 (922) | 26.5 (227) | 0.030 |
| Hemoglobin (g/dl) | 13.76 (1.80) | 13.37 (1.71) | | <0.001 | 13.61 (1.80) | 13.51 (1.65) | 0.104 |
| Serum Albumin (g/l) | 38.7 (4.1) | 37.8 (4.5) | | <0.001 | 38.3 (4.4) | 38.4 (3.7) | 0.757 |
| Heart rate (bpm) | 70.6 (12.1) | 70.3 (12.2) | | 0.375 | 70.7 (12.1) | 69.1 (12.3) | <0.001 |
| Current smoker | 17.7 (508) | 13.7 (294) | | <0.001 | 16.9 (696) | 11.9 (106) | <0.001 |
| Alcohol intake (≥ 3 times per week) | 20.4 (584) | 17.1 (363) | | 0.003 | 19.1 (782) | 18.6 (165) | 0.773 |
| Education |  |  | | <0.001 |  |  | <0.001 |
| ≤9 years | 48.2 (1354) | 62.9 (1325) | |  | 53.1 (2145) | 61.3 (534) |  |
| 10 years | 30.5 (855) | 25.9 (546) | |  | 29.3 (1183) | 25.0 (218) |  |
| >10 years | 21.3 (598) | 11.2 (236) | |  | 17.7 (715) | 13.7 (119) |  |

Data are mean (SD) for continuous variables and percentages (count) for categorical variables. T-test for continuous variables, Wilcoxon rank-sum test for UACR, chi² test for categorical variables. Missing values in following variables (number of missings): BMI (57), atrial fibrillation (12), valvular heart disease (42), anemia & hemoglobin (145), serum albumin (1), heart rate (49), current smoker (12), alcohol intake (28), education (101)
