# Supplementary material for: Heart Failure in a Cohort of Patients with Chronic Kidney Disease: The GCKD Study
Source: PLoS One. 2015 Apr 13;10(4):e0122552. doi: 10.1371/journal.pone.0122552 (PMC4395150; doi:10.1371/journal.pone.0122552)
Supplement: S2 Table — (DOCX) [file pone.0122552.s003.docx]

**S2 Table: Composition of the Gothenburg score in the GCKD study stratified by gender**

|  | Overall | Men | Women |
| --- | --- | --- | --- |
| *Cardiac score* |  |  |  |
| Positive score | 58% | 57% | 61% |
| Coronary heart disease | 20% | 25% | 13% |
| Angina pectoris | 9% | 9% | 10% |
| Edema | 40% | 35% | 48% |
| Dyspnea at night | 13% | 12% | 15% |
| Atrial fibrillation | 9% | 11% | 7% |
| Pulmonary rales | not obtained in GCKD | | |
| *Dyspnea on exertion* | 43% | 38% | 50% |
| *HF-medication* | 33% | 37% | 28% |
| Gothenburg stage |  |  |  |
| 0 | 42% | 43% | 39% |
| 1 (only positive cardiac score) | 15% | 16% | 15% |
| 2 (positive cardiac score and  dyspnea on exertion) | 17% | 13% | 23% |
| 2 (positive cardiac score and  HF-medication) | 10% | 12% | 7% |
| 3 (positive cardiac score and  dyspnea on exertion and  HF-medication) | 16% | 16% | 16% |
| Gothenburg HF  (stage 2 or 3) | 43% | 41% | 46% |
