# Supplementary material for: Heart Failure in a Cohort of Patients with Chronic Kidney Disease: The GCKD Study
Source: PLoS One. 2015 Apr 13;10(4):e0122552. doi: 10.1371/journal.pone.0122552 (PMC4395150; doi:10.1371/journal.pone.0122552)
Supplement: S3 Table — (DOCX) [file pone.0122552.s004.docx]

**S3 Table: Multivariable adjusted analyses of factors associated with Gothenburg HF among patients without self-reported HF (n=3,789)**

|  | Patients without self-reported heart failure | | |
| --- | --- | --- | --- |
|  | OR | 95% CI | *P* |
| eGFR (ml/min/1.73 m²) |  |  |  |
| ≥90 | 0.90 | 0.59-1.36 | 0.615 |
| 60-89 | 1.01 | 0.80-1.27 | 0.926 |
| 45-59 (reference) | ref | ref | ref |
| 30-44 | 1.18 | 0.99-1.41 | 0.067 |
| <30 | 1.85 | 1.39-2.46 | <0.001 |
| UACR (mg/g) (reference: <30) | ref | ref | ref |
| 30-299 | 0.80 | 0.66-0.96 | 0.018 |
| ≥300 | 0.94 | 0.77-1.16 | 0.583 |
| Age (5 year intervals) | 1.10 | 1.06-1.15 | <0.001 |
| Male gender | 0.58 | 0.49-0.68 | <0.001 |
| Diabetes mellitus | 1.58 | 1.33-1.86 | <0.001 |
| Hypertension | 1.75 | 1.19-2.58 | 0.005 |
| Valvular heart disease | 1.78 | 1.34-2.36 | <0.001 |
| BMI (kg/m²) | 1.10 | 1.09-1.12 | <0.001 |
| Sleep apnea | 2.07 | 1.57-2.73 | <0.001 |
| Anemia | 1.41 | 1.18-1.68 | <0.001 |
| Education (reference: ≤9 years) | ref | ref | ref |
| 10 years | 0.85 | 0.71-1.02 | 0.073 |
| >10 years | 0.64 | 0.51-0.81 | <0.001 |
| Serum albumin (g/l) | 0.95 | 0.93-0.96 | <0.001 |
| Heart rate (bmp) | 1.00 | 0.99-1.01 | 0.742 |
| Current smoker | 0.97 | 0.79-1.20 | 0.784 |
| Alcohol intake (≥ 3 times per week) | 0.90 | 0.73-1.10 | 0.282 |

Of 4126 observations, values were missing in BMI (47), valvular heart disease (33), anemia (114), education (83), heart rate (45), current smoker (11) and alcohol intake (24).
