# Supplementary material for: Heart Failure in a Cohort of Patients with Chronic Kidney Disease: The GCKD Study
Source: PLoS One. 2015 Apr 13;10(4):e0122552. doi: 10.1371/journal.pone.0122552 (PMC4395150; doi:10.1371/journal.pone.0122552)
Supplement: S4 Table — (DOCX) [file pone.0122552.s005.docx]

**S4 Table: Multivariable adjusted analysis of factors associated with HF restricted to patients recruited because of an eGFR <60 ml/min/1.73m² (n=4,278)**

|  | Gothenburg HF | |  | Self-reported HF | |  |
| --- | --- | --- | --- | --- | --- | --- |
|  | OR | 95% CI | *P* | OR | 95% CI | *P* |
| eGFR (ml/min/1.73 m²) |  |  |  |  |  |  |
| ≥90 | 1.93 | 0.99-3.76 | 0.053 | 0.68 | 0.20-2.31 | 0.532 |
| 60-89 | 1.08 | 0.88-1.34 | 0.455 | 1.14 | 0.88-1.49 | 0.330 |
| 45-59 (reference) | ref | ref | ref | ref | ref | ref |
| 30-44 | 1.18 | 1.01-1.38 | 0.042 | 1.11 | 0.91-1.35 | 0.297 |
| <30 | 1.67 | 1.29-2.16 | <0.001 | 1.28 | 0.95-1.72 | 0.101 |
| UACR (mg/g) (reference: <30) | ref | ref | ref | ref | ref | ref |
| 30-299 | 0.83 | 0.70-0.98 | 0.025 | 0.89 | 0.73-1.08 | 0.223 |
| ≥300 | 0.89 | 0.74-1.08 | 0.251 | 0.71 | 0.56-0.90 | 0.005 |
| Age (5 year intervals) | 1.14 | 1.09-1.18 | <0.001 | 1.15 | 1.09-1.22 | <0.001 |
| Male gender | 0.65 | 0.56-0.76 | <0.001 | 1.01 | 0.84-1.21 | 0.911 |
| Diabetes mellitus | 1.65 | 1.42-1.92 | <0.001 | 1.61 | 1.34-1.92 | <0.001 |
| Hypertension | 1.90 | 1.31-2.76 | 0.001 | 1.84 | 1.07-3.17 | 0.027 |
| Valvular heart disease | 2.57 | 2.05-3.23 | <0.001 | 3.94 | 3.15-4.92 | <0.001 |
| BMI (kg/m²) | 1.10 | 1.09-1.12 | <0.001 | 1.04 | 1.03-1.06 | <0.001 |
| Sleep apnea | 2.24 | 1.76-2.86 | <0.001 | 2.13 | 1.68-2.71 | <0.001 |
| Anemia | 1.42 | 1.21-1.68 | <0.001 | 1.06 | 0.87-1.28 | 0.592 |
| Education (reference: ≤9 years) | ref | ref | ref | ref | ref | ref |
| 10 years | 0.88 | 0.75-1.04 | 0.135 | 0.96 | 0.78-1.17 | 0.683 |
| >10 years | 0.69 | 0.56-0.85 | 0.001 | 1.01 | 0.79-1.31 | 0.910 |
| Serum albumin (g/l) | 0.96 | 0.94-0.97 | <0.001 | 1.00 | 0.98-1.03 | 0.795 |
| Heart rate (bmp) | 0.99 | 0.99-1.00 | 0.752 | 0.99 | 0.99-0.99 | 0.022 |
| Current smoker | 0.96 | 0.79-1.17 | 0.709 | 1.01 | 0.78-1.30 | 0.967 |
| Alcohol intake (≥ 3 times per week) | 0.98 | 0.82-1.17 | 0.824 | 1.00 | 0.81-1.25 | 0.968 |

Of 4,661 observations, values were missing in BMI (53), valvular heart disease (39), anemia (132), serum albumin (1), education (93), heart rate (47), current smoker (11) and alcohol intake (26).
